# Pathological features of West Nile and Usutu virus natural infections in wild and domestic animals and in humans: A comparative review

**DOI:** 10.1016/j.onehlt.2023.100525

**Published:** 2023-03-10

**Authors:** Gianfilippo Agliani, Giuseppe Giglia, Eleanor M. Marshall, Andrea Gröne, Barry H.G. Rockx, Judith M.A. van den Brand

**Affiliations:** a Division of Pathology, Faculty of Veterinary Medicine, Utrecht University, Utrecht, the Netherlands; b Department Viroscience, Erasmus University Medical Center, Rotterdam, the Netherlands; c Dutch Wildlife Health Centre (DWHC), Utrecht University, Utrecht, The Netherlands

**Keywords:** Mosquito-borne, Arbovirus, Flaviviruses, Pathology, Zoonoses, One Health

## Abstract

Mosquito-borne flaviviruses are emerging pathogens with zoonotic potential. Due to the recent climate and environmental changes, they are spreading across Europe, becoming a major threat for public and veterinary health. West Nile virus (WNV) and Usutu virus (USUV) are arboviruses that are responsible for multiple disease outbreaks in different species of birds, reptiles, and mammals, including humans. This review reports and compares the clinical signs as well as the gross and microscopic pathological features during natural infection with WNV and USUV in wild and domestic animals, as well as in humans. The main objective of this comparative review is to delineate the common features and the specific differences that characterize WNV- and USUV-induced diseases in each group of species and to highlight the main gaps in knowledge that could provide insight for further investigation on the pathogenesis and neurovirulence of these viruses.

## 1 Introduction

Emerging mosquito-borne flaviviruses are a major threat to human and animal health worldwide, in part due to the continued geographical expansion of their vector species [1]. West Nile Virus (WNV) and Usutu Virus (USUV) belong to the family Flaviviridae, genus Flavivirus, and are responsible for multiple outbreaks of disease in animals and humans spreading across Europe and North America alongside with climate changes [[2], [3], [4], [5]]. USUV isolates are currently classified into eight lineages, clustered into the African (AF1, -2, -3) and the European group (EU1, -2, -3, -4, -5) [6]. WNV isolates can be classified in at least eight lineages. Pathogenic strains belong to lineage 1, that include also Kunjin virus previously considered as a separate virus, and lineage 2 [7].

In mammals, tropism for the central nervous system (CNS) is a main feature of West Nile virus infection, characterized by the occurrence of encephalitis or encephalomyelitis, with or without meningeal involvement [3]. In contrast to what is commonly observed in mammals, in avian species, the involvement of the CNS is part of a severe systemic disease that often involves also other organs. Lesions associated with USUV infection are only reported in birds and, like WNV, affect multiple organs. Characteristics of WNV and USUV associated disease have been reported in the last decades in numerous studies, but a collection and comparison of common features in the same and different species is yet to be carried out. The aim of this paper is to give a general overview of the pathology during WNV and USUV infections in both wildlife, domestic animals, and in humans, focusing and delineating the main traits that characterize the disease for each virus, highlighting possible pathogenetic insights and main gaps in knowledge, that should represent topics for further investigations.

## 2 Transmission cycle and host range of WNV and USUV

WNV and USUV are both characterized by a similar enzootic transmission cycle that includes several species of mosquitoes as vectors and birds as amplifying hosts. Humans and other mammals act as incidental dead-end hosts as they do not develop high levels of viremia to maintain the transmission cycle.

WNV has been isolated from several species of mosquitoes worldwide, but the most relevant of these as vectors in the transmission cycle belong all to the genus *Culex* [8]. USUV has been isolated from *Culex pipiens*, which is considered the main vector, and its competence as vector has also been demonstrated under laboratory conditions together with *Cx. neavei* and *Cx. quinquefasciatus* [6].

WNV and USUV infections have been identified in a wide range of bird species, both clinically healthy animals in the context of active surveillance campaigns, as well as in diseased or dead bird species (listed in Table 1, Table 2). Among them, American crows (*Corvus brachyrhynchos*) [9] are considered highly susceptible to WNV infection while Eurasian blackbirds (*Turdus merula*) and great grey owls (*Strix nebulosa*) are highly susceptible to USUV [6]. In addition to infection in birds, WNV infects incidental hosts, mainly humans and horses, and sporadically other species listed in Table 3. USUV infection has been detected in various mammals including horses [10], dogs [11], bats [12], squirrels[13], wild boars, wild ruminants [14], lizards [15] and humans [16]; however, lesions are only reported in birds.

**Table 1:** Avian species reported as susceptible to WNV disease development.

Order	Species
*Passeriformes*	House sparrow *(Passer domesticus)* [17]
	Yellow-billed magpie *(Pica nuttalli)* [18]
	Blue jay *(Cyanocitta cristata)* [19]
	Loggerhead shrike *(Lanius ludovicanus)* [20]
	American crow (*Corvus brachyrhynchos*) [9]
	Fish crow (*Corvus ossifragus*) [9]
	Black-billed magpies (*Pica pica)* [9]
*Strigiformes*	Barred owl *(Strix varia)* [21]
	Short-eared owl *(Asio flammeus)* [21]
	Great-horned owl *(Bubo virginianus)* [21,22]
	Snowy owl (Bubo scandiacus) [9,21]
Accipitriformes	Northern goshawk (Accipiter gentilis) [[22], [23], [24]]
	Sharp-shinned hawk *(Accipiter striatus)* [22]
	Cooper’s hawk *(Accipiter cooperi)* [22,25,26]
	Red-tailed hawk *(Buteo jamaicensis)* [22,[25], [26], [27]]
	Golden eagle *(Aquila chrysaetos)* [28]
	Spanish imperial eagle *(Aquila adalberti)* [29]
	Bald eagle *(Haliaeetus leucocephalus)* [9,28]
*Falconiformes*	Gyrfalcon *(Falco risticulus)* [30]
	Prairie falcon *(Falco mexicanus)* [22]
	Peregrine falcon *(Falco peregrinus)* [22]
*Anseriformes*	Mallard *(Anas platyrhynchos)* [31]
	Canadian goose *(Branta canadensis)* [31]
	Lesser scaup *(Aythya affinis)* [32]
	Bronze-winged duck (*Anas specularis*) [9]
*Galliformes*	Common pheasant *(Phasianus cholchicus)* [33]
	Turkey *(Meleagris gallopavo)* [34]
	Himalayan Impeyan pheasant (*Lophophorus impeyanus*) [9]
	Blyth's tragopan (*Tragopan blythi*) [9]
*Ciconiiformes*	Chilean flamingo (*Phoenicopterus chilensis*) [9]
	Black-crowned night heron (*Nycticorax nycticorax*) [9]
*Pelicaniformes*	Guanay cormorant (*Phalacrocorax bougainvillea*) [9]
*Charadriiformes*	Laughing gull (*Larus atricilla*) [9]
*Psittaciformes*	Slender-billed conure *(Enicognathus leptorhynchus)* [35]
	Moluccan cockatoo *(Cacatua moluccensis)* [35]
	Australian king parrot *(Alisterus scapularis)* [35]
	Princess of Wales parakeet *(Polytelis alexandrae)* [35]
	Red rump parakeet *(Psephotus haematonotus)* [35]
	Swainson’s lorikeet *(Trichoglossus moluccanus)* [35]
	Nanday conure *(Aratinga nenday)* [35]
	Black-headed caique *(Pionites melanocephalus)* [35]
	Indian ring neck parakeet *(Psittacula krameri)* [35]
	Turquoise parrot *(Neophema pulchella)* [35]
	Crimson rosella *(Platycercus elegans)* [35]
	Scarlet-chested parakeet *(Neophema splendida)* [35]
	Eastern rosella *(Platycercus eximius)* [35]
	Derbyan parakeet *(Psittacula derbiana)* [35]
	Sun conure *(Aratinga solstitialis)* [35]
	Western rosella *(Platycercus icterotis)* [35]
	Goldie’s lorikeet *(Psitteuteles goldiei)* [35]

**Table 2:** Avian species reported as susceptible to USUV disease development.

Order	Species
*Passeriformes*	Barn swallow *(Hirundo rustica)* [36]
	Blackbird *(Turdus merula)* [[36], [37], [38], [39]]
	Common starling *(Sturnus vulgaris)* [40]
	Domestic canary *(Serinus canaria)* [40]
	Eurasian bullfinch *(Pyrrhula pyrrhula)* [41]
	Eurasian jay *(Garrulus glandarius)* [40]
	European robin *(Erithacus rubecula)* [42]
	Fieldfare *(Turdus pilaris)* [43]
	House sparrow *(Passer domesticus)* [40]
	Song thrush *(Turdus philomelos)* [40]
*Strigiformes*	Boreal owl *(Aegolius funerius)* [39]
	Great grey owl *(Strix nebulosa)* [40]

**Table 3:** Non-avian species reported as susceptible to WNV disease development.

	Species
Equids	Horse *(Equus caballus)* [[44], [45], [46]]
Ruminants	Sheep *(Ovis aries)* [47]
	Alpaca *(Vicugna pacos)* [48]
	Reindeer (*Rangifer tarandus*) [49]
	White-tailed deer (*Odocoileus virginianus*) [50]
Canids	Wolf (*Canis lupus*) [51]
	Dog (*Canis lupus familiaris*) [51]
Marine mammals	Harbor Seal (*Phoca vitulina*) [52]
	Killer whale (*Orcinus orca*) [53]
Rodents	Eastern Fox Squirrels (*Sciurus niger*) [54]
Marsupials	Virginia opossum (*Didelphis Virginiana*) [55]
Non-human primates	Barbary Macaque (*Macaca sylvanus*) [56]
Reptiles	American alligators (*Alligator mississippiensis*) [57,58]

## 3 Pathogenesis of WNV- and USUV-associated disease in animals and humans

Disease associated with WNV and USUV infection has been reported in several animal species. Birds infected with USUV or WNV usually show a multisystemic disease involving the central nervous system, liver, spleen, heart and kidney [9,59]. In addition, in raptors the eye is reported as one of the main target organs during WNV natural infection [26]. However, whilst incidental hosts may show pathology in numerous organ systems, clinical disease primarily manifests with neurological symptoms. The pathogenesis and dissemination of WNV has been widely studied in mice models and avian natural hosts, although many of its aspects are still to be elucidated. Virus is transmitted by mosquito bite, although, based on identification of virus antigen in the neurons of enteric ganglia, a possible oral transmission has also been proposed [59]. In the skin, WNV is thought to replicate in keratinocytes and Langerhans dendritic cells, the latter migrate to draining lymph nodes, from which a primary viremia starts with subsequent infection of peripheral organs such as the spleen [60]. Neuroinvasion is one of the most relevant and investigated aspects of WNV infection. Regardless of the transmission mechanism of the virus, two overarching routes of WNV neuroinvasion have been proposed: transneural and hematogenous. Transneural pathways include transport along neurons of the periphery or olfactory tract directly into the brain. Hematogenous invasion could occur transcellularly via transcytosis of virus across the blood-brain barrier (BBB), or paracellularly as a result of inflammatory disruption of the BBB resulting in invasion of virus, either free in the blood or trafficked within infected immune cells acting as a so called ‘Trojan horse’ [60]. Knowledge on pathogenesis of USUV is still lacking, but due to the close relatedness with WNV, similar pathways of neuroinvasion are hypothesized.

In general, the main pathologic features of WNV and USUV infection include necrosis and inflammation. The pathogenesis behind the cell death during WNV and USUV infection is still unknown. For WNV two overarching mechanisms are thought to be involved: (1) a direct cytopathic effect of the virus on the cells and (2) the host antiviral immune response [61]. It has been shown, using both *in vivo* and *in vitro* models, that WNV can induce cell death in neurons through different mechanisms such as apoptosis [62] and pyroptosis [63]. The inflammation induced by WNV is mainly represented by infiltration of lymphocytes, plasma cells and histiocytes and is suggested to be triggered by the production of cytokines such as IL-1β, -6, -8, and tumor necrosis factor (TNF)-α in the tissue [64]. Innate immunity during WNV infection is mediated by several pathogen recognition receptors (PRRs), RNA binding produces downstream activation of pathways that ultimately induce expression of IFN I and III molecules with direct or indirect antiviral functions. The adaptive immune response against flaviviruses is either mediated by antibodies produced by B-lymphocytes or T-cell mediated cytotoxicity. Regarding T-cell immunity CD4+ cells provide help for antibody response and sustain CD8+ mediated viral clearance. CD8+ cells mediated immunopathology is described in mammals and is mainly related to aberrant cytokine production and or cytolytic activity [65]. In this review we report the clinical features, lesions and viral antigen distribution associated with WNV and USUV infection in the main target organs of different host species and summarize these findings in Table 4, Table 5, Table 6, Table 7, Table 8.

**Table 4:** Comparison of histologic lesions in the nervous system of birds infected with WNV or USUV.

	Passerine birds		Nocturnal raptors		Diurnal raptors		Waterfowls		Game birds		Psittacine birds	
	WNV	USUV	WNV	USUV	WNV	USUV	WNV	USUV	WNV	USUV	WNV	USUV
Central Nervous System												
Endothelial cell degeneration	++	+	+	+	+	NI	++	-	-	NI	-	NI
Vasculitis	++	+	+	+	+	NI	++	-	-	NI	-	NI
Neuronal necrosis	-	+	++	+	++	NI	-	-	-	NI	-	NI
Gliosis	+	+	++	+	++	NI	++	-	+	NI	-	NI
Meningoencephalitis	+	+	++	+	++	NI	++	-	+	NI	++	NI
Myelitis	-	NE	+	NE	+	NI	+	NE	-	NI	+	NI

Peripheral Nervous System												
Ganglion neuritis	-	+	+	NE	+	NI	-	-	-	NI	-	NI
Peripheral/optic perineuritis	-	NE	+	NE	+	NI	-	-	-	NI	-	NI

“++” main feature**;** “+” present (not main feature)**;** “–“absent**;** “NI” infection not reported in the animal group**;** “NE” not evaluated**.**

In the present table, the term “Game birds” is referred to the species belonging to the order *Galliformes* as listed in Table 1

**Table 5:** Comparison of histologic lesions in extra neurologic organs in bird infected with WNV or USUV.

	Passerine birds		Nocturnal raptors		Diurnal raptors		Waterfowls		Game birds		Psittacine birds	
	WNV	USUV	WNV	USUV	WNV	USUV	WNV	USUV	WNV	USUV	WNV	USUV
Liver												
Hepatic necrosis	++	++	++	++	-	NI	+	-	-	NI	+	NI
Hepatitis	++	++	++	++	+	NI	++	-	+	NI	++	NI

Hematopoietic System												
Splenic necrosis	++	++	++	-	++	NI	+	-	+	NI	+	NI
Arterial fibrinoid necrosis	-	-	-	-	+	NI	-	-	-	NI	-	NI
Lymphoid depletion	-	-	++	-	+	NI	+	-	-	NI	-	NI
Splenitis	-	-	-	-	-	NI	-	-	-	NI	++	NI
Bone marrow necrosis	++	-	-	-	-	NI	-	-	-	NI	-	NI
Bursal atrophy	+	-	-	-	-	NI	+	-	-	NI	-	NI

Cardiovascular system												
Myocardial necrosis	+	++	++	-	+	NI	++	-	++	NI	++	NI
Myocarditis	++	+	++	-	++	NI	+	-	++	NI	++	NI
Vascular/perivascular lesions	+	-	-	-	-	NI	-	-	-	NI	-	NI

Eye												
Pectenitis/choroiditis/uveitis	NE	NE	++	NE	++	NI	NE	NE	NE	NI	+	NI
Retinal atrophy	NE	NE	-	NE	+	NI	NE	NE	NE	NI	-	NI
Optic discitis	NE	NE	-	NE	+	NI	NE	NE	NE	NI	-	NI
Optic nerve gliosis	NE	NE	-	NE	+	NI	NE	NE	NE	NI	-	NI
Hemorrhages	NE	NE	-	NE	+	NI	NE	NE	NE	NI	-	NI

Respiratory system												
Laryngotracheitis	-	NE	+	NE	+	NI	-	NE	+	NI	-	NI
Lung necrosis	+	-	-	-	-	NI	-	-	-	NI	-	NI
Pulmonary hemorrhages	+	-	+	-	+	NI	-	-	-	NI	-	NI
Capillary thrombosis	-	-	+	-	+	NI	-	-	-	NI	-	NI
Interstitial pneumonia	+	+	-	+	-	NI	-	-	+	NI	-	NI

Kidney												
Tubular/glomerular necrosis	++	+	++	-	+	NI	+	-	-	NI	+	NI
Tubulo-interstitial nephritis	++	+	+	+	++	NI	-	-	-	NI	++	NI
Hemorrhages	-	-	-	-	-	NI	+	-	-	NI	-	NI

Skin												
Dermatitis	-	+	+	-	+	NI	-	-	-	NI	-	NI
Vasculitis	-	+	-	-	-	NI	-	-	-	NI	-	NI

Skeletal muscle												
Myonecrosis	NE	NE	+	NE	-	NI	NE	-	-	NI	+	NI
Myositis	NE	NE	-	NE	+	NI	NE	-	+	NI	+	NI

Gastrointestinal system												
Hemorrhages	+	-	-	-	-	NI	-	-	-	NI	-	NI
Mucosal necrosis	++	-	+	-	-	NI	-	++	-	NI	+	NI
Lymphoid tissue necrosis	+	-	-	-	-	NI	-	-	+	NI	-	NI
Pharyngitis/esophagitis	-	-	+	-	-	NI	-	-	-	NI	-	NI
Pro-/ventriculitis	-	+	-	-	+	NI	-	-	+	NI	+	NI
Enteritis	-	+	+	+	+	NI	-	++	-	NI	+	NI
Caecal tonsillitis	+	-	-	-	-	NI	-	-	-	NI	-	NI
Pancreatic necrosis	+	-	-	-	-	NI	+	-	+	NI	+	NI
Pancreatitis	-	-	+	-	+	NI	-	-	+	NI	+	NI

Endocrine system												
Thyroiditis	-	-	+	NE	+	NI	-	NE	-	NI	-	NI
Thyroidal hemorrhages	-	-	+	NE	-	NI	++	NE	-	NI	-	NI
Thyroidal necrosis	-	-	-	NE	-	NI	-	NE	-	NI	-	NI
Parathyroid necrosis	-	NE	+	NE	-	NI	-	NE	-	NI	-	NI
Adrenal hemorrhages	-	NE	-	NE	-	NI	+	NE	-	NI	+	NI
Adrenalitis	-	NE	-	NE	-	NI	+	NE	-	NI	+	NI
Genital system												
Oophoritis	-	NE	+	NE	-	NI	-	NE	-	NI	-	NI
Granulosa cells necrosis	-	NE	+	NE	-	NI	-	NE	-	NI	-	NI

“++” main feature**;** “+” present (not main feature)**;** “– “absent**;** “NI” infection not reported in the animal group**;** “NE” not evaluated**.**

In the present table, the term “Game birds” is referred to the species belonging to the order *Galliformes* as listed in Table 1

**Table 6:** Comparison of presence of virus antigen by immunohistochemistry in birds infected with WNV or USUV.

	Passerine birds		Nocturnal raptors		Diurnal raptors		Waterfowls		Game birds		Psittacine birds	
	WNV	USUV	WNV	USUV	WNV	USUV	WNV	USUV	WNV	USUV	WNV	USUV
Central Nervous System												
Neurons	+	+	+	NE	+	NI	+	NE	+	NI	+	NI
Glial cells	+	+	+	NE	+	NI	+	NE	-	NI	-	NI
Endothelial cells	+	+	+	NE	+	NI	-	NE	-	NI	-	NI

Peripheral Nervous System												
Ganglion neurons	-	+	+	NE	+	NI	-	NE	-	NI	+	NI
Gangliar glial cells	-	+	+	NE	+	NI	-	NE	-	NI	+	NI

Liver												
Hepatocytes	-	+	+	NE	+	NI	+	NE	-	NI	+	NI
Kupffer cells	+	+	+	NE	+	NI	+	NE	-	NI	+	NI
Endothelial cells	-	+	+	NE	+	NI	+	NE	-	NI	+	NI
Infiltrating macrophages	-	+	+	NE	+	NI	+	NE	-	NI	+	NI

Spleen												
Mononuclear phagocytes	+	+	-	NE	-	NI	+	NE	-	NI	+	NI
Vascular smooth muscle cells	-	+	+	NE	-	NI	-	NE	-	NI	+	NI
Fibrocytes	-	+	+	NE	-	NI	-	NE	-	NI	+	NI

Heart												
Cardiomyocytes	+	+	-	NE	-	NI	+	NE	-	NI	-	NI
Fibrocytes	+	+	-	NE	-	NI	-	NE	-	NI	-	NI
Endothelial cells	+	+	-	NE	-	NI	-	NE	-	NI	-	NI
Vascular smooth myocytes	+	+	-	NE	+	NI	-	NE	-	NI	-	NI

Respiratory system												
Pneumocytes	-	+	-	NE	-	NI	+	NE	-	NI	+	NI
Smooth myocytes	-	+	-	NE	-	NI	+	NE	-	NI	+	NI
Fibrocytes	+	+	NE	NE	NE	NI	NE	NE	NE	NI	+	NI
Tracheal chondrocytes	+	+	NE	NE	NE	NI	NE	NE	NE	NI	+	NI
Alveolar macrophages	+	+	+	NE	-	NI	+	NE	-	NI	+	NI
Circulating monocytes	+	+	+	NE	-	NI	+	NE	-	NI	+	NI

Kidney												
Tubular epithelial cells	-	+	-	NE	+		-	NE	-	NI	-	NI
Macrophages	+	+	-	NE	-	NI	-	NE	-	NI	-	NI
Endothelial cells	-	+	+	NE	-	NI	-	NE	-	NI	-	NI
Fibrocytes	-	+	+	NE	-	NI	-	NE	-	NI	-	NI

Skin												
Fibrocytes	+	+	-	NE	-	NI	-	NE	-	NI	-	NI
Macrophages	+	+	-	NE	-	NI	-	NE	-	NI	-	NI
Epidermal keratinocytes	+	+	-	NE	-	NI	-	NE	-	NI	-	NI
Follicular keratinocytes	+	+	-	NE	-	NI	-	NE	-	NI	-	NI
Feather pulp	+	+	-	NE	-	NI	-	NE	-	NI	-	NI

Skeletal muscle												
Myocytes	NE	NE	+	NE	+	NI	NE	NE	-	NI	+	NI

Gastrointestinal system												
Mucosal epithelial cells	+	+	+	NE	+	NI	+	NE	-	NI	-	NI
Infiltrating macrophages	-	+	+	NE	+	NI	-	NE	-	NI	-	NI
Fibrocytes	-	+	+	NE	+	NI	-	NE	-	NI	-	NI
Smooth myocytes	-	+	+	NE	+	NI	-	NE	-	NI	-	NI

Endocrine system												
Thyroid follicular epithelium	-	+	+	NE	+	NI	+	NE	-	NI	+	NI
Adrenocortical cell	-	+	+	NE	+	NI	+	NE	-	NI	+	NI
Fibrocytes	-	+	+	NE	+	NI	+	NE	-	NI	+	NI
Infiltrating macrophages	-	+	+	NE	+	NI	+	NE	-	NI	+	NI

Eye												
Choroid	NE	NE	-	NE	+	NI	NE	NE	NE	NI	-	NI
Retinal neurons	NE	NE	-	NE	+	NI	NE	NE	NE	NI	-	NI
Pecten	NE	NE	-	NE	+	NI	NE	NE	NE	NI	-	NI
Pigmented epithelial cells	NE	NE	-	NE	+	NI	NE	NE	NE	NI	-	NI
Macrophages	NE	NE	-	NE	+	NI	NE	NE	NE	NI	-	NI
Melanocytes	NE	NE	-	NE	+	NI	NE	NE	NE	NI	-	NI

Gonads												
Thecal cells	+	+	+	NE	NE	NI	NE	NE	NE	NI	+	NI
Interstitial cells	+	+	+	NE	NE	NI	NE	NE	NE	NI	+	NI
Granulosa cells	+	+	+	NE	NE	NI	NE	NE	NE	NI	+	NI
Infiltrating macrophages	+	+	+	NE	NE	NI	NE	NE	NE	NI	+	NI
Testis germ cells	+	+	+	NE	NE	NI	NE	NE	NE	NI	+	NI

“+” IHC positive cells**;** “-” IHC negative cells**;** “NI” infection not reported in the animal group**;** “NE” not evaluated**.**

In the present table, the term “Game birds” is referred to the species belonging to the order *Galliformes* as listed in Table 1

**Table 7:** Comparison of histologic lesions in organs of humans, other mammals and reptiles infected with WNV.

	Equids	Ruminants	Carnivores	Marine mammals	Rodents	Marsupials	Non-human primates	Reptiles	Humans
Central Nervous System									
Neuronal necrosis	+	+	+	+	+	-	-	-	++
Endothelial cells degeneration	-	-	-	+	-	-	-	-	-
Vasculitis	-	-	-	++	+	-	-	-	-
Meningoencephalitis	++	++	++	++	++	+	++	++	++
Myelitis	++	+	++	+	-	-	-	++	++
Gliosis	+	+	+	+	+	-	++	-	++

Peripheral Nervous System									
Ganglion neuritis	-	NE	NE	NE	-	NE	NE	NE	NE
Perineuritis	-	NE	NE	NE	-	NE	NE	NE	NE

Liver									
Hepatic necrosis	+	-	-	NE	+	+	-	-	NE
Perivascular/periportal hepatitis	-	-	-	NE	-	+	-	-	NE

Spleen									
Splenic necrosis	-	-	-	NE	-	-	NE	+	NE
Lymphoid depletion	+	-	-	NE	-	-	NE	+	NE
Splenitis	-	-	-	NE	-	-	NE	+	NE

Heart									
Myocardial necrosis	-	-	-	NE	++	++	NE	+	+
Myocarditis	+	-	++	NE	++	++	NE	+	+

Respiratory system									
Laryngotracheitis	-	NE	NE	NE	-	NE	NE	-	NE
Epithelial necrosis	-	-	-	NE	-	-	-	-	NE
Hemorrhages	-	-	-	NE	-	-	-	-	NE
Capillary thrombosis	-	-	-	NE	-	-	-	-	NE
Interstitial pneumonia	+	-	-	NE	+	-	-	+	NE

Kidney									
Tubular/glomerular necrosis	-	-	-	NE	-	-	+	++	-
Tubulo-interstitial nephritis	-	-	-	NE	++	+	+	+	+

Skin									
Lymphohistiocytic proliferative syndrome	-	NE	NE	NE	-	NE	NE	++	-

Skeletal muscle									
Hemorrhages	-	-	+	NE	-	-	NE	-	NE
Myonecrosis	-	-	+	NE	-	++	NE	-	NE
Myositis	-	-	-	NE	-	++	NE	-	NE

Gastrointestinal system									
Hemorrhages	+	-	-	NE	-	-	NE	-	NE
Mucosal necrosis	-	-	-	NE	-	-	NE	+	NE
Enteritis	-	-	-	NE	-	-	NE	+	NE

Endocrine system									
Adrenal necrosis	NE	NE	-	NE	-	NE	NE	+	NE
Adrenalitis	NE	NE	-	NE	-	NE	NE	+	NE

Eye									
Choroiditis/uveitis	NE	NE	NE	NE	NE	NE	NE	-	+
Retinal atrophy	NE	NE	NE	NE	NE	NE	NE	-	+
Optic discitis	NE	NE	NE	NE	NE	NE	NE	-	+
Optic nerve gliosis	NE	NE	NE	NE	NE	NE	NE	-	-
Hemorrhages	NE	NE	NE	NE	NE	NE	NE	-	+

“++” main feature**;** “+” present (not main feature)**;** “–“absent**;** “NI” infection not reported in the animal group**;** “NE” not evaluated**.**

A detailed list of the species included within the animal groups reported in the present table is provided on Table 3

**Table 8:** Comparison of presence of virus antigen by immunohistochemistry in humans, other mammals and reptiles infected with WNV.

	Equids	Ruminants	Carnivores	Marine mammals	Rodents	Marsupials	Non-human primates	Reptiles	Humans
Central Nervous System									
Neurons	+	+	+	+	NE	NE	+	NE	+
Glial cells	+	+	+	+	NE	NE	+	NE	-
Endothelial cells	+	-	-	-	NE	NE	-	NE	-

Heart									
Cardiomyocytes	NE	-	+	NE	NE	NE	NE	NE	NE
Fibrocytes	NE	-	-	NE	NE	NE	NE	NE	NE
Endothelial cells	NE	-	-	NE	NE	NE	NE	NE	NE
Vascular smooth muscle cells	NE	-	-	NE	NE	NE	NE	NE	NE

Skin									
Fibrocytes	NE	NE	NE	NE	NE	NE	NE	NE	-
Macrophages	NE	NE	NE	NE	NE	NE	NE	NE	+
Epidermal keratinocytes	NE	NE	NE	NE	NE	NE	NE	NE	-
Follicular keratinocytes	NE	NE	NE	NE	NE	NE	NE	NE	-
Feather pulp	NE	NE	NE	NE	NE	NE	NE	NE	-
Endothelial cells	NE	NE	NE	NE	NE	NE	NE	NE	+

Endocrine system									
Thyroid follicular epithelium	NE	NE	NE	NE	NE	NE	NE	NE	NE
Adrenocortical cell	NE	-	+	NE	NE	NE	NE	NE	NE
Fibrocytes	NE	-	NE	NE	NE	NE	NE	NE	NE
Infiltrating macrophages	NE	-	NE	NE	NE	NE	NE	NE	NE

“+” IHC positive cells**;** “-” IHC negative cells**;** “NI” infection not reported in the animal group**;** “NE” not evaluated**.** Investigation of the presence of the viral antigen through immunohistochemistry in non-avian species is rarely reported. Positivity is present in only few organs listed in the table. A more extensive overview is provided in the supplementary materials.

## 4 Clinical features of WNV- and USUV-associated disease in animals

Clinical disease in birds infected with WNV and USUV presents as non-specific (e.g., immobility, apathy, ruffled feather) and neurological signs (ataxia, paresis, tremors, torticollis, inability to fly and seizures) [24,59], furthermore vision loss is reported for WNV natural infection in diurnal raptors [26]. WNV infection of incidental host species is most often asymptomatic but presents mainly as neurological disease in the small percentage of symptomatic cases. Specifically, in patients around 80% of the cases are asymptomatic. Most symptomatic patients present with a mild, self-limiting febrile disease called West Nile fever (WNF), often accompanied by headache, myalgia, lethargy and gastrointestinal complaints. Approximately 1% of human infections results in severe neurological involvement, called West Nile neuroinvasive disease (WNND), with a large range of manifestations such as generalized weakness [16,66], hyperreflexia [67], transient paralysis of limbs [68], facial paralysis [69], dysgeusia, aphasia [66], ataxia and dysmetria [16]. In the few reported human clinical cases described so far, USUV infections present with mild symptoms such as rash and fever, however, evidence of USUV neuroinvasive disease has been reported in several countries [16,66,67,70], mainly concerning patients with contributing risk factors. Nevertheless, pathology data are lacking.

## 5 Pathological features of WNV-associated disease in animals

The description of WNV-associated disease lesions will focus on the most commonly affected organs.

### 5.1 Central and peripheral nervous system

CNS disease is the main feature of WNV infection in mammals and birds. Macroscopical lesions including hemorrhages, and malacia are only occasionally reported [3,44,47,71,72]. Microscopically, parenchymal lesions, such as non-suppurative meningoencephalitis with lymphohistiocytic and lymphoplasmacytic perivascular cuffs, neuronal necrosis with formation of glial nodules, encephalomalacia and gliosis are reported [3,9,18,28,[47], [48], [49], [50], [51],^53^,[55], [56], [57],[73], [74], [75], [76], [77]], as well as vascular lesions such as acute necrotizing vasculitis, degeneration of endothelial cells and hemorrhages [9,17,53] (Table 4). In most species, lesions are mainly located in the brainstem and cerebellum [3,31,32,34,35,[47], [48], [49], [50], [51], [52], [53],[55], [56], [57],^72^,^76^,^77^]. However, cerebrum can also be a target for WNV, as reported in some birds [35]. A multifocal lymphoplasmcytic myelitis of variable severity has been reported in birds [32,72], some mammals and in reptiles [[44], [45], [46], [47],^49^,^51^,^57^,^74^,^76^,^79^]. WNV antigen is seen in the cerebrum and cerebellum of both birds and mammals, mainly in the cytoplasm of neurons [3,17,19,28,29,32,34,35,[47], [48], [49],^51^,^56^,^72^] and glial cells [3,17,19,28,29,32,51,72]. Endothelial cell positivity is reported in the brain of birds [19,28,72] and horses [3]. Lesions of the peripheral nervous system are reported only in raptors. Heterophilic or lymphoplasmacytic ganglion neuritis or perineuritis [24,72] occurs in the sciatic nerve [72], the myenteric, cervical and periadrenal ganglia, and the epicardial nerves. WNV antigen in immunohistochemistry (IHC) is present in neurons or glial cells of the myenteric and submucosal ganglia [72] of raptors, rarely also in psittacine birds [35].

### 5.2 Liver

Hepatic gross lesions including hepatomegaly [35,57,72], tan discoloration [35,57] and petechiae [35] are reported in birds and reptiles. Microscopically, randomly distributed necrosis is the main finding [9,17,57,72], while a defined pattern of midzonal necrosis is reported in horses [46]. Birds [9,[18], [19], [20],^24^,^28^,^31^,^34^,^35^] and some mammals [55,77] show a lymphoplasmacytic hepatitis with periportal or random distribution. In reptiles, in addition to the previously mentioned more acute lesions, hepatitis is associated with ductular hyperplasia and fibrosis [57]. WNV antigen is reported in Kupffer’s cells [19,28,29,31,35], endothelial cells, hepatocytes and macrophages [28,29,31,32,72] of birds. Scattered positive macrophages are observed in squirrels [77].

### 5.3 Spleen

Grossly, splenomegaly and splenic necrosis are often observed in birds [17,18,20,24,31,35,72] and reptiles [57]. Microscopically, the most common finding is necrosis with a periarteriolar arrangement [9,19,72], followed by red and white pulp hyperplasia and lymphoid apoptosis [9,19]. Occasionally, splenitis with a mixed inflammatory cell population, including heterophils [35,54,72] is seen in birds and horses [46] and in alligators [57]. As for vascular lesions, fibrino-necrotizing arteritis is reported. Substantial presence of WNV antigen is found in the cells of the mononuclear phagocytes system [19,20,31,35], smooth muscle cells of the arteries walls and fibrocytes in the capsule [35,72].

### 5.4 Heart

Grossly, myocardial pallor and epicardial and subepicardial petechiae are observed [31,32,35,72]. Microscopically, myocardial necrosis is the main feature in birds, marsupials and reptiles [17,55,57], while in other species it is lymphohistiocytic myocarditis [9,[17], [18], [19], [20],^51^,^54^,^55^,^77^]. Vascular and perivascular inflammation is occasionally observed in the large vessels of the heart of passerines. In the avian cardiocirculatory system, high presence of WNV antigen is present in cardiomyocytes [17,19,31,32], interstitial cells [19], macrophages [19], endothelial cells [17] and vascular smooth muscle cells [17,19,26]. Positive cardiomyocytes are reported also in Virginian opossum (*Didelphis virginiana*) [55].

### 5.5 Eye

Ocular lesions have only been described in raptors and remain under investigated in other relevant avian groups such as Passerine birds**.** Gross lesions include intraocular hemorrhage [24,26], discoloration of the retina, vitreous humor opacity [26], corneal erosion [24], and areas of opacity in the fundus [27,28]. Microscopically, lymphoplasmacytic pectenitis is most frequently reported, followed by iridocyclitis and keratoconjunctivitis [72]. Chorioretinal lesions have been classified based on their severity and include degeneration and disarray of the retinal architecture and lymphoplasmacytic infiltration of the choroid, optic disc and optic nerve [24,27,28]. WNV antigen is seen in choroid and pecten in passerine birds without lesions, while in raptors it is commonly observed in retinal neurons, pigmented epithelial cells, macrophages in the iris and melanocytes in the pecten [[26], [27], [28]].

### 5.6 Kidney

Regarding the kidney, macroscopically, renomegaly and pale appearance are reported in birds [72]. Microscopically, tubular epithelial necrosis and [17,19] tubulo-interstitial lymphohistiocytic nephritis are the main histologic finding [18] in birds, rodents, non-human primates and reptiles [28,35,54,56,57,72]. Suppurative interstitial nephritis is observed in marsupials [55]. The presence of WNV antigen is reported in birds and in squirrels [24,26,28,29,31,34,72] targeting tubular epithelial cells [25,54], infiltrating macrophages [19,20,54], endothelial cells of glomerular tufts, and interstitial cells [72].

### 5.7 Other organs

Lesions and viral antigen distribution associated with WNV infection are reported in detail in Table 5, Table 6 for birds and Table 7, Table 8 for mammals and reptiles.

## 6 Pathological features of USUV-associated disease in birds

The description of USUV-associated disease lesions will focus on the most commonly affected organs.

### 6.1 Nervous system

In passerine birds and nocturnal raptors, the lesions are described as a non-suppurative encephalitis with neuronal degeneration and necrosis, satellitosis, neuronophagia, glial nodules and lymphohistiocytic perivascular cuffs in the cerebrum and brainstem [[37], [38], [39],^41^,^59^,^80^]. In the cerebellum, aggregates of microglia around necrotic neurons within the granular layer, called glial shrubberies, are noticed [[37], [38], [39],^81^]. In some species [59,82], there is additional endothelial cell swelling and vasculitis. Virus antigen has been observed in neurons, glial cells and endothelial cells [[36], [37], [38], [39],^83^].

### 6.2 Liver

The liver is a commonly affected organ in birds. Variable degrees of hepatomegaly are reported macroscopically [[36], [37], [38], [39],^41^,^80^,^81^,^84^]. Microscopically, this is associated with hepatic necrosis and mononuclear cell infiltration [[37], [38], [39],^41^,^84^]. Virus antigen is reported mainly in Kupffer cells, endothelial cells and circulating mononuclear cells, less commonly hepatocytes are also positive [[36], [37], [38], [39],^41^,^81^,^84^,^85^].

### 6.3 Spleen

Splenomegaly is one of the major macroscopic findings associated with USUV-infection [38,80,84]. Microscopically, zonal necrosis centered in the sheathed arteries in association with histiocytic hyperplasia is seen in raptors and passerines [[37], [38], [39],^41^,^84^]. The same lesions are also hallmarks of avian malaria, caused by highly prevalent haemoprotozoan infection in passerine birds [86] possibly also in co-infection with USUV. Regarding virus antigen distribution is seen in capsule spindle cells, endothelial cells and mononuclear cells [37,41,84].

### 6.4 Heart

In passerines [[37], [38], [39],^81^] multifocal pallor of the myocardium is only described in birds co-infected with *Plasmodium* spp. Microscopically, myocardial necrosis and lymphoplasmacytic myocarditis are reported [37]. In nocturnal raptors, hydropericardium is seen in animals co-infected with *Plasmodium* spp. in association with myocardial degeneration [[36], [37], [38]]. Virus antigen is commonly detected in cardiomyocytes and in mononuclear cells infiltrating, as well as in vascular endothelial cells [[37], [38], [39],^81^].

### 6.5 Kidney

In birds, macroscopical evidence of nephromegaly and pallor [37,38] are associated microscopically with perivascular lympho-histiocytic infiltrations and tubular necrosis [[37], [38], [39],^41^,^82^,^84^]. Virus antigen is seen in tubular epithelial cells, glomerular capillaries and mononuclear cells infiltrating in the interstitium and in vascular endothelium.

### 6.6 Other organs

Lesions associated with USUV infection have been observed in several organs of the respiratory and gastrointestinal system. Furthermore, site-specific hyperkeratosis of peri cloacal skin is observed in blackbirds [38,59]. Associated histological lesions and viral antigen distribution are reported in detail in Table 5, Table 6.

## 7 Pathological features of WNV-associated disease in humans

There are no fatal human cases of USUV associated disease described in literature, therefore only pathology resulting from WNV-associated disease is described in humans.

### 7.1 Nervous system

Histological findings of WNND vary depending on the severity and presentation of disease. In general, non-supportive encephalitis, characterized by lymphohistiocytic perivascular cuffs, and neuronal necrosis with neuronophagia and microglial nodules are observed in the midbrain, pons, medulla, substantia nigra and cerebellum [69,[87], [88], [89], [90], [91], [92], [93]]. Gliosis is also observed in the lumbar region [88,90] of the spinal cord. Presence of viral antigen is seen in neurons and Purkinje cells often associated with foci of inflammation, except in severely immunocompromised patients who also show extensive WNV staining independent of inflammatory lesions [69,88,91,93].

### 7.2 Heart

Macroscopically, cardiomegaly has been observed, and microscopic examination showed myocytic necrosis and lymphohistiocytic myocarditis [94,95] with scarring.

### 7.3 Eye

Ocular manifestations such as uveitis, optic neuritis, occlusive vasculitis and chorioretinitis are reported, as well as isolated cases of chorioretinitis [[96], [97], [98], [99]], neuritis [96,97,100] and vitritis [96,99], most often associated with meningitis and encephalitis. However, cases involving only the eye suggest that ocular symptoms can also occur in absence of descending infection from the brain to the ocular nerves. Neuronal atrophy was observed in the inner retinal layer, however no retinal tissue was available for IHC to confirm or discount presence of virus.

### 7.4 Kidney

In severely immunocompromised patients, virus antigen and inflammation has been reported in the kidney [69,91], but how such findings may contribute to disease and correlate with clinical presentations during acute infection and convalescence is yet to be fully determined.

### 7.5 Other organs

Histopathological changes have been observed also in the skin, including hemorrhages and thrombi within the dermis, as well as perivascular inflammatory infiltrates. WNV antigen was identified within the cytoplasm of perivascular infiltrates, as well as the vascular endothelium of the small dermal vessels. Lesions and viral antigen distribution associated with WNV infection in humans are reported in detail in Table 7, Table 8.

## 8 Discussion

Lesions associated with WNV infection are reported in a wide range of animals and in humans. In USUV infection, despite evidence of infection in mammals and disease reported in humans, pathological data are available only for birds. In order to extrapolate the known mechanisms and tropism involved in WNV pathogenesis to the lesser-known mechanisms of USUV pathogenesis, WNV and USUV-associated disease can be compared according to the type of lesions, distribution of viral antigen by IHC and clinical presentation.

### 8.1 WNV and USUV neurologic disease

For WNV, in all hosts, the main pattern of lesions in the CNS is characterized by lymphohistiocytic meningoencephalomyelitis and neuronal loss [101,102]. The same pattern is observed in birds naturally infected with USUV, though lesions are not reported in the spinal cord, probably due to lack of investigation in this organ. The pathogenetic mechanisms behind the cellular damage and subsequent functional impairment associated with WNV and USUV infection are still to be completely elucidated [61]. WNV can induce cell damage either directly or indirectly through the immune reaction of the host. *In vivo* and *in vitro* experimental studies have shown that WNV can induce programmed cell death, with a more severe effect if it occurs in non-renewing cell populations, such as neurons [62,63,102]. Additionally, WNV infection can trigger an immune reaction by inducing the production of several inflammatory mediators; this promotes neuroinflammation and appears to be a main factor driving WNV-induced neuronal damage [64]. At the same time, virus-induced immune response is suggested to have a negative impact on neurotransmission, this has been highlighted by the upregulation of pleiotropic genes functioning at several level of the immune-neural-synaptic axis in a Non-Human Primate (NHP) model of WNV-ND [103]. Nevertheless, how much cell injury can be attributed to viral cytopathology and how much to the inflammatory response is still not known.

Distribution of viral antigen in the CNS is similar in all hosts but does not always reflect the localization of the lesions. The uniformity of virus distribution between hosts suggests the involvement of receptor molecules highly preserved among animal species; these are thought to belong to the C-type lectins and glycans families, although specific molecules still need to be identified [104]. The discrepancy between viral antigen distribution and the location of lesions supports the theory of an immunologic component in the pathogenesis of WNV disease. In horses has been observed either presence of viral antigen in absence of microscopic changes or low amount of antigen compared to lesion severity, both findings suggest that the pathological changes may follow the virus distribution in time and may therefore be seen even without the presence of virus antigen [35,44]. Lesions and viral tropism in the CNS associated with USUV infection are reported only in birds and are comparable to what is observed in birds infected with WNV. The similarity between the two viruses in the histopathological patterns of lesions and viral antigen distribution in the CNS provides insightful indications to drive future investigations on the pathogenesis of USUV-associated disease and, at the same time, can represent a diagnostic challenge raising the need to develop specific techniques for the differentiation of the two agents. This is difficult due to the high chance of cross-reaction between closely related flaviviruses belonging to the same serocomplex, as in this case.

Neuronal dysfunction or loss in specific areas of the brain can be linked to the clinical signs observed in both humans and animals. Involvement of cerebellar and vestibular nuclei of the brainstem that are responsible for the control of motor activity, may cause incoordination of movements, such as ataxia and dysmetria. Facial paresis, observed in humans, could be linked to the affection of the facial nucleus in the brainstem and damage to the dopaminergic neurons of the substantia nigra could cause tremors due to loss of modulation on motor function. Impairment of the motor neurons of the ventral horns of the spinal cord can be associated with clinical evidence of paraparesis [105]. Seizures are commonly associated with damage to cerebral cortex, that for WNV infection has been observed only in psittacine. Nevertheless, seizures could be additionally explained by the more widespread effect of IL-6 and TNF-α produced by macrophages in response to viral infection in the CNS. These cytokines increase neural hyperexcitability by disrupting the neuronal excitation/inhibition balance [106]. The same symptoms have been observed during USUV infection in humans, for which neuropathology is still to be investigated. Since the close relatedness of the two viruses, the comparison of similar neurological symptoms observed in USUV neuroinvasive disease and in WNND, may suggest a similar neurolocalization and pattern of the lesions for USUV in humans.

### 8.2 WNV and USUV extra neurologic disease

**I**n addition to the changes in the CNS, necrosis and lymphoplasmacytic inflammation mainly affecting heart, liver, spleen, kidney and eye, represent a typical pattern of lesions during WNV and USUV infection in birds. In the accidental hosts, extra neurological lesions are less commonly reported, even though they can sometimes be a prominent feature of the infection, such as myocarditis in canids and marsupials or renal necrosis in reptiles. Similar pathologic changes are observed in multiple organs in course of avian malaria, posing this disease in the differential diagnosis of flaviviral infection[86]. Furthermore, due to the high prevalence of haemoprtozoa in birds, co-infection with either WNV or USUV is common and needs further investigation to understand its implications in the pathology and pathogenesis of these infections.

Viral antigen in both definitive and accidental animal host is widely distributed in several extra neurological tissue and cell types also in albescence of microscopic changes sustaining the previously discussed immune-mediated tissue damage. The differences in lesions and viral antigen distribution between avian and mammalian species could be due to (1) differences in the host immune reaction: in humans a widespread distribution of WNV antigen in several non-CNS organs has been observed in immunocompromised patients showing WNND [91]; or (2) the levels of viremia: it is known that birds develop higher levels of viremia compared to other hosts such as humans in which WNV is rarely isolated from patients with WNND [91].

Although vascular changes such as vasculitis and fibrinoid necrosis are common additional findings in WNV infection, a histopathological pattern, in which vascular lesions are predominant, is observed in house sparrow nestlings and is also reported as main finding in a killer whale that died acutely without premonitory signs. In both the birds and the mammal, the pattern was associated with minimal inflammation that can indicate a rapidly fatal course of the infection in these animals [17,53]. In a fatal human case of fulminant hemorrhagic fever associated with WNV infection there was widespread endothelial positivity for WNV antigen in several organs including the CNS [107]. This evidence poses the hemorrhagic disease in the spectrum of possible clinic-pathological manifestations of WNV infection in both animals and humans.

Both WNV and USUV antigen has been highlighted by IHC in the cytoplasm of neurons of the myenteric plexuses in raptors and passerine birds with evidence of ganglion neuritis, that are never reported in mammals and humans, probably due to lack of systematic investigation of the GI tract in these hosts. Contribution of flaviviral infection to the development of intestinal dysmotility has been suggested in a mouse model, posing these viruses among the possible causes of GI dysmotility syndrome also in humans, highlighting the necessity of extending pathological and immunohistochemical investigation to other organs such as the GI tract in the accidental hosts [108].

### 8.3 Reservoir potential and surveillance implications

Clinical presentation of WNV disease in birds has been experimentally studied in several species, while data on USUV-associated disease mainly comes from passive surveillance on wild birds. Therefore, insight into the clinical progression of USUV-associated disease is lacking. Blackbirds and great grey owls are considered particularly susceptible species to USUV infection mainly due to the high mortality rate observed during the outbreaks of the disease. However, highly susceptible species might not be the best reservoir of the infection in nature. To define a species as reservoir, several indicators are required; among them there is the susceptibility to natural infection[109] and the ability to maintain a sufficient level of viremia long enough to infect a new mosquito. Animals that die acutely are less likely to spread the infection and animals that may chronically carry the infection with a high viremic level are difficult to identify through surveillance on wildlife due to the ethical concerns and practical difficulties posed by repeated sampling.

Currently, tissues (found dead birds), blood and swabs (live caught birds) are collected as samples for monitoring and surveillance. However, these samples are often difficult to obtain and invasive for live birds. Determining the viral tropism in tissues through IHC provides insights in the locations of the lesions and the infected cell types in order to improve surveillance in dead and live birds. As was shown in previous studies, USUV and WNV antigen have been demonstrated in mature feather follicles of naturally infected birds [23,59], suggesting feathers to be an easy, effective and non-invasive method for testing in monitoring and surveillance.

## 9 Future perspective

The effect of climate change and increasing urbanization is changing the dynamics of mosquito-borne diseases, making them more likely to spread across countries where they were not commonly observed before. With the outbreaks of WNV and USUV in birds and the presence of WNV and USUV in mosquitos, mammals and humans, it is important to be prepared for increased incidence of human cases. Therefore, a One Health approach to improve preparedness strategies to deal with future outbreaks is needed. The identification of the host range of these viruses can give important information to improve monitoring and surveillance, including an early warning system in case of an outbreak with reliable diagnostic methods and relevant therapeutics and perhaps more preventive measures. For early warning systems the use of sentinel birds is crucial in detecting and monitoring mosquito-borne flaviviruses. To determine the appropriate sentinel species but also to develop therapeutic and preventive measures, knowledge on the clinical and pathological features as well as the pathogenesis in amplifying and incidental hosts for WNV and USUV is essential.

## Funding

This publication is part of the project ‘Preparing for Vector-Borne Virus Outbreaks in a Changing World: a One Health Approach’ (NWA.1160.1S.210), which is (partly) financed by the Dutch Research Council (NWO).

## CRediT authorship contribution statement

**Gianfilippo Agliani:** Conceptualization, Writing – original draft, Writing – review & editing. **Giuseppe Giglia:** Conceptualization, Writing – original draft, Writing – review & editing. **Eleanor M. Marshall:** Writing – original draft, Writing – review & editing. **Andrea Gröne:** Writing – review & editing. **Barry H.G. Rockx:** Writing – review & editing. **Judith M.A. van den Brand:** Writing – review & editing, Supervision.

## Declaration of Competing Interest

The authors declare that they have no known competing financial interests or personal relationships that could have appeared to influence the work reported in this paper.

## Data Availability

No data was used for the research described in the article.

## Appendix A Supplementary data

Supplementary material Image 1
