# Supplementary material for: Pathological features of West Nile and Usutu virus natural infections in wild and domestic animals and in humans: A comparative review
Source: One Health. 2023 Mar 10;16:100525. doi: 10.1016/j.onehlt.2023.100525 (PMC10288044; doi:10.1016/j.onehlt.2023.100525)
Supplement: Supplementary file 1 — Supplementary material [file mmc1.docx]

**Comparison of presence of virus antigen by immunohistochemistry in humans, other mammals and reptiles infected with WNV.**

|  | Equids | Ruminants | Carnivores | Marine mammals | Rodents | Marsupials | Non-human primates | Reptiles | Humans |
| --- | --- | --- | --- | --- | --- | --- | --- | --- | --- |
| Peripheral Nervous System | | | | | | | | | |
| Gangliar neurons | NE | NE | NE | NE | NE | NE | NE | NE | NE |
| Gangliar glial cells | NE | NE | NE | NE | NE | NE | NE | NE | NE |
| Liver | | | | | | | | | |
| Hepatocytes | NE | - | NE | NE | NE | NE | - | NE | NE |
| Kuppfer’s cells | NE | - | NE | NE | NE | NE | - | NE | NE |
| Endothelial cells | NE | - | NE | NE | NE | NE | - | NE | NE |
| Infiltrating macrophages | NE | - | NE | NE | NE | NE | - | NE | NE |
| Spleen | | | | | | | | | |
| Mononuclear phagocytes | NE | - | - | NE | NE | NE | NE | NE | NE |
| Vascular smooth myocytes | NE | - | - | NE | NE | NE | NE | NE | NE |
| Fibrocytes | NE | - | - | NE | NE | NE | NE | NE | NE |
| Respiratory system | | | | | | | | | |
| Pneumocytes | NE | - | NE | NE | NE | NE | - | NE | NE |
| Smooth myocytes | NE | - | NE | NE | NE | NE | - | NE | NE |
| Fibrocytes | NE | - | NE | NE | NE | NE | - | NE | NE |
| Tracheal chondrocytes | NE | NE | NE | NE | NE | NE | NE | NE | NE |
| Alveolar macrophages | NE | - | NE | NE | NE | NE | - | NE | NE |
| Circulating monocytes | NE | - | NE | NE | NE | NE | - | NE | NE |
| Kidney | | | | | | | | | |
| Tubular epithelial cells | NE | - | - | NE | NE | NE | - | NE | NE |
| Macrophages | NE | - | - | NE | NE | NE | - | NE | NE |
| Endothelial cells | NE | - | - | NE | NE | NE | - | NE | NE |
| Fibrocytes | NE | - | - | NE | NE | NE | - | NE | NE |
| Skeletal muscle | | | | | | | | | |
| Myocytes | NE | NE | NE | NE | NE | NE | NE | NE | NE |
| Gastrointestinal system | | | | | | | | | |
| Mucosal epithelial cells | NE | - | NE | NE | NE | NE | NE | NE | NE |
| Infiltrating macrophages | NE | - | NE | NE | NE | NE | NE | NE | NE |
| Fibrocytes | NE | - | NE | NE | NE | NE | NE | NE | NE |
| Smooth myocytes | NE | - | NE |  | NE | NE | NE | NE | NE |
| Eye | | | | | | | | | |
| Choroid | NE | NE | NE | NE | NE | NE | NE | NE | NE |
| Retinal neurons | NE | NE | NE | NE | NE | NE | NE | NE | NE |
| Pecten | NE | NE | NE | NE | NE | NE | NE | NE | NE |
| Pigmented epithelial cells | NE | NE | NE | NE | NE | NE | NE | NE | NE |
| Macrophages | NE | NE | NE | NE | NE | NE | NE | NE | NE |
| Melanocytes | NE | NE | NE | NE | NE | NE | NE | NE | NE |
| Gonads | | | | | | | | | |
| Perifollicular macrophages | NE | NE | NE | NE | NE | NE | NE | NE | NE |
| Thecal cells | NE | NE | NE | NE | NE | NE | NE | NE | NE |
| Interstitial cells | NE | NE | NE | NE | NE | NE | NE | NE | NE |
| Granulosa cells | NE | NE | NE | NE | NE | NE | NE | NE | NE |
| Infiltrating macrophages | NE | NE | NE | NE | NE | NE | NE | NE | NE |
| Testis germ cells | NE | NE | NE | NE | NE | NE | NE | NE | NE |

“+” IHC positive cells**;** “-” IHC negative cells**;** “NI” infection not reported in the animal group**;** “NE” not evaluated**.**
